# Platelet thrombus formation in patients with end-stage renal disease before and after hemodialysis as measured by the total thrombus-formation analysis system

**DOI:** 10.1007/s11255-022-03184-7

**Published:** 2022-04-02

**Authors:** Branka P. Mitic, Zorica M. Dimitrijevic, Kazuya Hosokawa, Tatjana P. Cvetkovic, Milan V. Lazarevic, Danijela D. Tasic, Andriana Jovanovic, Nina Jancic, Tamara Vrecic, Anna Ågren, Håkan Wallen

**Affiliations:** 1 Faculty of Medicine, University of Nis, Nis, Serbia; 2 Clinical Center Nis, Clinic for Nephrology, Nis, Serbia; 3 Department for ECC and Hemostasis, Clinical Center Nis, Clinic for Cardiac Surgery, Nis, Serbia; 4 Research Institute, Fujimori Kogyo Co., Ltd., Yokohama, Kanagawa Japan; 5 Division of Cardiovascular Medicine, Department of Clinical Sciences, Danderyd Hospital, Karolinska Institutet, Stockholm, Sweden; 6 Department of Molecular Medicine and Surgery, Karolinska Institutet, Stockholm, Sweden

**Keywords:** End-stage renal disease, Hemodialysis, Microchip flow chamber system, Platelet function

## Abstract

**Background:**

Patients with end-stage renal disease (ESRD) receiving hemodialysis (HD) often experience bleeding. However, mechanisms behind this bleeding tendency are incompletely understood but may involve platelet dysfunction. We, therefore, studied platelet-dependent thrombus formation in flowing whole blood inside a microchip coated with collagen, and its association with circulating von Willebrand factor (VWF).

**Methods:**

Blood samples were obtained in 22 patients before and after HD. The area under the 10 min flow pressure curve in a microchip (AUC10) reflecting total platelet thrombogenicity was measured, using the Total Thrombus-formation Analysis System (T-TAS01). AUC10 < 260 indicates platelet dysfunction. VWF activity and antigen in plasma were also assayed.

**Results:**

VWF levels were moderately elevated and increased further after HD (*P* < 0.01 or lower). In contrast, AUC10 before and after HD was < 260 in 17/22 patients and < 130 in 15/22 patients, with no statistically significant difference in pre- vs post-HD measurements, indicating reduced platelet thrombogenicity, but with some variability as 5/22 patients showed normal platelet responsiveness. AUC10 and VWF activity or antigen levels in plasma were not correlated, either before or after HD.

**Conclusions:**

Most ESRD patients display moderate-to-severe platelet dysfunction as assessed by shear-induced platelet-dependent thrombus formation with T-TAS01. HD does not influence platelet function despite HD-induced elevations in VWF. T-TAS01 should be further evaluated as a tool in the assessment of bleeding risk in patients on HD.

**Graphical abstract:**

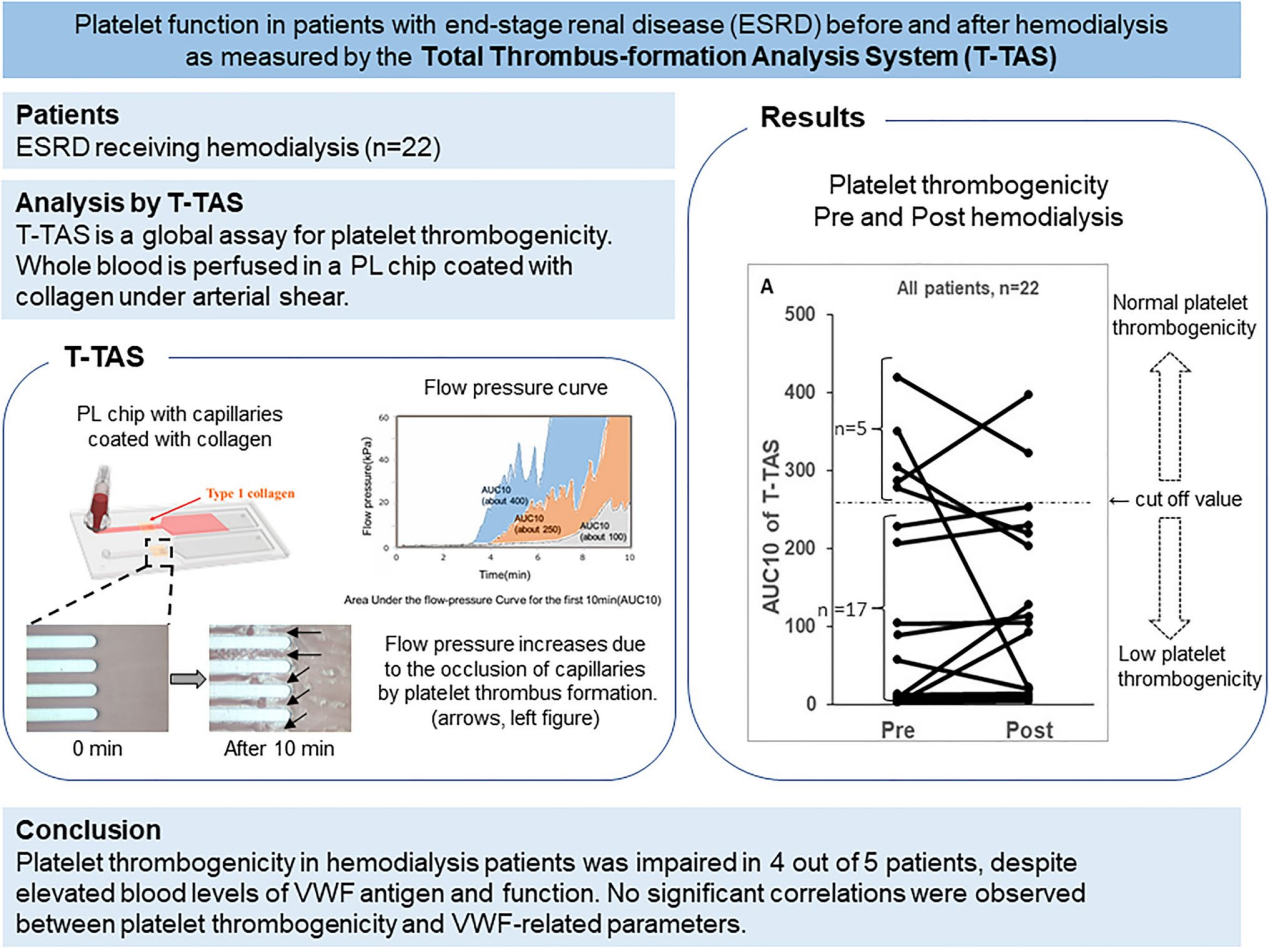

## Introduction

End-stage renal disease (ESRD) patients, particularly those treated with hemodialysis (HD), encounter a variety of hemostatic disorders ranging from bleeding diathesis to thrombotic tendencies and vascular complications. Bleeding complications in ESRD, which vary in different studies between 2.1 and 16.1 per 100 person years [1], may be due to abnormalities in platelet function, impairment in the platelet–vessel wall interaction, and due to anticoagulants administered during HD [2]. Notably, HD itself also has pro-inflammatory and pro-coagulatory effects, and increases oxidative stress, although such effects may be reduced by aggressive dialysis-based removal of so-called middle molecules [3] and use of more biocompatible dialysis membranes [4].

The impact of HD on platelet function and primary hemostasis has been investigated rather extensively including studies on effects on platelet aggregation, platelet glycoprotein receptor (GP) exposure, and quantity and function of VWF, the latter being a molecule central to primary hemostasis. Results on effects of HD are, however, inconsistent and confounds our overall understanding of the features of primary hemostasis in hemodialysis patients. There are numerous studies assessing bleeding and thrombotic propensities in different patient populations, including those with ESRD, using different platelet function methods. Light transmission aggregometry and other platelet aggregation tests, including Multiplate analyzer and VerifyNOW, which all depend on the addition of soluble platelet agonists, such as collagen, arachidonic acid or ADP to assess platelet reactivity in platelet-rich plasma or whole blood, have been used in various study settings [5, 6]. Other methods used are e.g., flow cytometry analysis of platelet glycoprotein receptor expression and functional tests of VWF enabling the evaluation of other platelet function aspects including platelet adhesion [7–10]. However, due to the complexity and heterogeneity of patients with ESRD undergoing HD, it could be hypothesized that methods that assess global aspects of platelet function may be advantageous. It is furthermore possible that techniques which measure platelet function in whole blood under flow are advantageous as they include interacting red blood cells and rheology in the assessment of hemostasis adhesion [11]. In this respect, the Total Thrombus Formation Analysis System (T-TAS01) is of interest. This method has been developed to assess platelet function in whole blood under flow. The method uses a microchip flow chamber system and measures platelet thrombus formation in whole blood under arterial shear flow conditions (1500 s^−1^) in a microchip (PL-chip) coated with type I collagen [12]. In the present study, we used T-TAS01 to study shear flow-based platelet thrombogenicity in ESRD patients before and after HD.

## Materials and methods

This single-center observational cohort study was approved by the Ethics Committee of Nis University Hospital (approval number 39454, November 1, 2019). The study was performed in accordance with the declaration of Helsinki. Oral and written informed consent was obtained from all participants. Inclusion criteria were ongoing hemodialysis (HD) and acceptance to participate in the study. Exclusion criteria were chronic hepatitis C or B infection, active infection, presence of central venous catheter or arteriovenous graft, known vascular access dysfunction and/or need for antithrombotic drugs (including antiplatelet agents).

Patients were included during September 2020. All patients were dialyzed through an arteriovenous fistula of the upper limb using high-flux steam sterilized polysulphone dialysers (Polyflux Gambro, Hechingen, Germany), and unfractionated heparin was used as anticoagulant during HD.

### Blood sampling

Blood samples were taken with a 14 G needle through the arteriovenous fistula and collected before heparin infusion at the start of hemodialysis as well as at the end of HD session, and was immediately transferred into EDTA tubes (Vacusera, Turkey), benzylsulfonyl-d-Arg-Pro-4-amidinobenzylamide (BAPA)-tubes (Fujimori Kogyo Co., Ltd, Tokyo, Japan), and sodium citrate tubes (Vacusera, Turkey) for measurements of complete blood cell counts (CBC), T-TAS PL-chip, and other biochemical tests (see below), respectively.

### Laboratory tests

Blood cells were analyzed on Nihon Kohden Hematology Analyzer while biochemical data were measured on Siemens Dimension RXL Max Chemistry Analyzer. Coagulation tests, including measurements of prothrombin time-international normalized ratio (PT-INR), activated partial thromboplastin time (APTT), partial thromboplastin time (PTT) and fibrinogen, were performed with the semi-automated ACL Elite Pro coagulation analyzer (Instrumentation Laboratory Bedford USA).

VWF antigen, VWF activity, VWF ristocetin cofactor activity (RCo), and VWF collagen binding activity (CBA) were measured by automated chemiluminescent immunoassay, namely HemosIL AcuStar VWF:AG, VWF:ACT, VWF:RCo and VWF:CBA (Instrumentation Laboratory BIOKIT, S.A., Barcelona, Spain), respectively, using the AcuStar hemostasis testing system. The VWF Activity kit was measured with a latex particle-enhanced immunoturbidimetric assay for quantifying VWF Activity in plasma.

### Platelet thrombus formation analysis

Platelet thrombus formation on a collagen surface was evaluated under flow conditions using T-TAS01 (Fujimori Kogyo, Japan). Immediately before measurement, 400 μL of whole blood anticoagulated with 50 μM BAPA, a synthetic dual inhibitor of factor Xa and thrombin, was mixed with 4 μl of 50,000 U/mL heparinase-I (final concentration; 500 U/mL) for 3 min at room temperature. After heparin neutralization, 330 μL of whole blood sample was perfused into a microchip coated with type-1 collagen (PL-chip) with a shear rate of 1500 s^−1^ [13]. Platelets adhered and aggregated on the collagen surface of the microchip channel, resulting in the formation of small platelet thrombi. The platelet thrombi gradually expanded and eventually occluded the microchip channel. Platelet thrombus formation was measured by the increase of the flow pressure inside the microchip. Platelet thrombogenicity was defined as the area under the flow pressure curve for 10 min, abbreviated as AUC10 [12]. A cut-off value for impaired platelet function has previously been defined as AUC10 < 260 s, and is based on measurements in 142 healthy volunteers [14]

### Statistical analysis

The study was exploratory and no formal power calculation was performed. The primary variable was platelet thrombogenicity expressed as AUC10. The AUC10 values are shown as median and IQR, or as mean ± SD. Statistically significant differences in AUC10 and VWF-related variables before versus after HD were determined using the Wilcoxon signed-rank test or paired t test, as appropriate. Spearman’s rank correlation coefficient (rs) was used to evaluate pairwise correlations between AUC10 and VWF-related variables. A *p* value of < 0.05 was considered statistically significant. All data were analyzed using JMP IN^®^ software (SAS Institute, Tokyo, Japan).

## Results

### Patient characteristics and general blood chemistry

We investigated 22 patients undergoing regular HD (18 men, 4 women, aged 66 ± 11 years). All patients were in a stable phase of disease and were hepatitis C-virus negative. The average duration on HD was 49 ± 31 months (range 6–111 months). All patients were free from anticoagulant or antiplatelet medication (aspirin, P_2_Y_12_ inhibitors or NSAIDs) during at least 14 days before the investigation. None had experienced any recent bleeding, thrombotic or inflammatory complication three months prior to the investigation.

The etiologies of ESRD were hypertensive nephropathy (*n* = 8), diabetic nephropathy (*n* = 6), autosomal dominant polycystic kidney disease (*n* = 2), tubulointerstitial nephritis (*n* = 2), chronic pyelonephritis (*n* = 1), glomerulonephritis (*n* = 1), and unknown (*n* = 2).

Duration of dialysis (4–4.5 h), blood (270–300 mL/min) and dialysate flow (500 mL/min) were prescribed to a *Kt*/*V* > 1.2. Unfractionated heparin was used at a dosage of 78 ± 17 U/kg. Eighteen out of 22 patients (82%) were receiving recombinant human erythropoietin beta, mean dose 5144 ± 4264 Units.

The hemoglobin and hematocrit values were 105 ± 23 g/L and 33 ± 4%, respectively. Platelet counts were 176 ± 48 × 10^9^/L, and PTT, APTT, and PT-INR were all within the reference ranges (107 ± 12%, 31 ± 6 s, and 1.03 ± 0.07, respectively), whereas plasma-fibrinogen was moderately elevated (5.6 ± 1.6 g/L).

### Platelet thrombus formation before and after hemodialysis

**Fig. 1:**
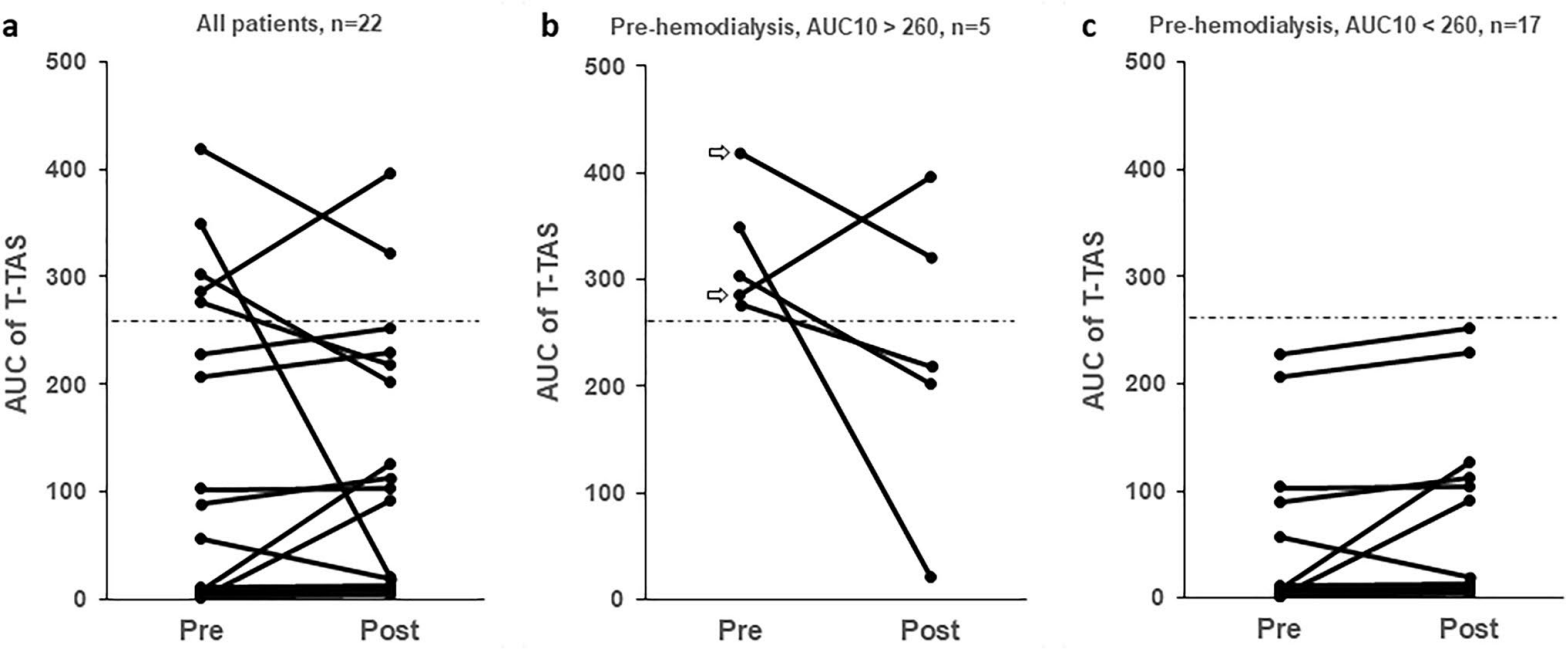
Platelet thrombogenicity before (pre) and after (post) hemodialysis in 22 patients with ESRD. AUC10 is the area under the flow pressure curve during 10 min building up when platelet thrombi occlude micro-capillaries in a chip. The hatched lines show cut-off to identify platelet dysfunction (AUC10 260). Data on all patients are shown in **a** (left), in patients with normal platelet function (above cut-off) in **b** (middle), and in patients with impaired platelet function (below cut-off) in **c** (right). In two patients with normal platelet function (**b**) the etiology for ESRD was diabetic nephropathy (indicated with arrows)

AUC10 was 10.25 [3.6–222.5] (median [IQR], range 2.3–419.1) before, and 20.3 [7.2–183.6] (range 4.5–396.1) after HD, respectively (*p* = 0.37; Fig. 1a). However, both the median and mean AUC10 values before and after HD were much lower than in a population of healthy US residents (*n* = 142, median 390.9, mean 381.0) [16].

In 17 out 22 patients (77%), AUC10 was below the cut-off value identifying patients with impaired platelet function (AUC10 < 260) [14], and in more than two-thirds of patients AUC was 50% below this cut-off (15 of 22, i.e. 68% of patients with AUC < 130).

To further study possible effects of HD per se, we performed a sub-group analysis in patients with preserved (AUC > 260) vs those with reduced platelet-dependent thrombus formation (AUC < 260) prior to the start of the HD procedure (Fig. 1b, c). In patients with preserved platelet-dependent thrombus formation (*n* = 5), the median AUC10 value decreased non-significantly after HD in 4/5 patients (AUC pre, 303.1 [286.1–349.4] vs post, 218.6 [202.5–321.5]) (*P* = 0.31; Fig. 1b). In the group of patients with impaired platelet thrombus formation already at start of HD, 15 of 17 patients showed a slight but non-significant increase in platelet thrombogenicity after HD (AUC pre, 5.9 [3.1–56.6] vs post, 11.3 [5.8–103.9], *n* = 17) (*P* = 0.07; Fig. 1c).

### Von Willebrand factor (VWF)

VWF antigen, and measures of VWF activity, i.e. VWF-RCo, VWF-CBA, and latex particle-based VWF activity are shown in Table 1. Mean values of VWF antigen and VWF-CBA were above the reference range before HD. Of note, VWF antigen and VWF activity increased after HD in all patients, VWF-RCo increased in 21 of 22 patients, and VWF-CBA increased in 19 of 22 patients.

**Table 1:** Plasma levels of von Willebrand factor (*n* = 22)

Von Willebrand factor (% of normal)	Before HD	After HD	*P* value
Antigen (Ag)	152 ± 53	183 ± 63	< 0.001
Ristocetin-cofactor activity (RCo)	137 ± 53	176 ± 65	< 0.01
Collagen-binding activity (CBA)	156 ± 58	195 ± 78	< 0.001
Latex-particle-based activity (Act)	163 ± 59	205 ± 82	< 0.001

Data are mean ± SD

*HD* hemodialysis

*P* values denote comparison before vs after (paired t test)

The VWF-RCo: VWF antigen ratio before and after HD were 1.02 ± 0.1 and 1.04 ± 0.1, respectively.

### Correlations between platelet thrombus formation and VWF

AUC10 before and after HD correlated fairly well (*r* = 0.77, *P* < 0.001), and all VWF-related parameters (before vs after HD) were also correlated (*r* = 0.74 or higher; *p* < 0.0001). In contrast, no significant correlations were found between absolute values of AUC10 and any VWF-related parameters or between changes in values (i.e. delta-values for differences between pre- and post-HD). This indicates that platelet thrombus formation under high shear, as measured by the collagen-coated PL-chip, is not importantly influenced by a moderate HD-induced increase in VWF.

## Discussion

We investigated platelet-dependent thrombogenicity in flowing whole blood before and after HD in 22 patients with ESRD, using a collagen-coated PL chip. The major findings are as follows: (1) Shear-based platelet thrombogenicity (AUC10) was reduced and below the cut-off value for platelet dysfunction in 17/22 of patients investigated; (2) HD did not enhance platelet thrombogenicity significantly; (3) platelet thrombogenicity was not related to VWF or its changes in plasma in response to HD, although all assessed aspects of VWF increased significantly following HD platelet thrombogenicity was not enhanced.

Problems with bleeding are common in patients with severe renal failure. A retrospective cohort study in more than 11 000 patients on HD found that 1 in 7 of patients will have a major bleeding within 3 years. It was concluded that improved risk stratification of HD patients with respect to bleeding is needed [15]. Furthermore, up to 50% of patients with renal failure have been described to suffer from bleeding of various type and degree [16]. It is interesting to note that we presently find impaired platelet function in 17/22 patients as assessed by the T-TAS01, using the cut-off established in a healthy control population [14]. All our patients were free from antiplatelet drugs. The mechanisms behind our findings are likely multifactorial and at present unclear. Of note, it should be acknowledged that numerous previous studies—summarized in Table 2—have investigated effects of HD on platelet function and VWF-related parameters. In this respect, it is of interest to note that platelet thrombus formation inside the PL-chip of T-TAS01 includes both platelet adhesion onto collagen and platelet aggregation, mechanisms that include interactions with VWF and fibrinogen on one side, and platelet glycoprotein (GP) receptors on the other [17, 18]. Blockade of the platelet GPIbα receptor (mediating platelet adhesion through VWF bound to collagen), or the platelet GPIIbIIIa receptor (mediating aggregation mainly through fibrinogen and/or VWF binding) effectively inhibit platelet thrombus formation in the PL-chip [19]. Based on these studies and previous studies showing reduced expression and/or availability of the GPIb receptor in ESRD patients [20, 21], it is possible that reduced GP-receptor availability is one explanation to our findings of reduced platelet thrombogenicity in the patients.

**Table 2:** Effects of hemodialysis on platelet function

	Chronic effects (pre-hemodialysis vs healthy control)	Transient effects (change in pre- vs post-hemodialysis)
Platelet aggregation	No significant difference in platelet aggregation (collagen/ADP) was observed between hemodialysis patients and the healthy control [5] SIPA was significantly reduced in hemodialysis patients [22]	No significant difference in platelet aggregation (collagen/ADP) was observed before and after hemodialysis [5] SIPA was further reduced after hemodialysis [28]
VWF-related parameters	VWF antigen and VWF Rco levels in hemodialysis patients were higher than those in the healthy control [9, 10, 23]	VWF antigen and VWF Rco levels were further elevated after hemodialysis [9, 23]
Platelet membrane glycoprotein (GP) expression	GPIb, GPIIbIIIa and P-selectin were reduced in hemodialysis patients compared to the healthy control [22, 23, 25]	GPIb, GPIIbIIIa and P-selectin of pre-hemodialysis were further reduced [22, 23]

Reduced GPIbα and increased VWF in HD patients have opposing effects on the initial platelet adhesion process. These counteracting effects transiently strengthen after HD [22, 23]. The effects of VWF- and GPIbα-changes may cancel out each other but may also produce large individual differences in the ability of primary hemostasis among HD patients as shown in our study.

Reduced expression or availability of the GPIIbIIIIa receptor can also contribute to our finding, an idea supported by previous studies in ESRD [8]. Decreased platelet adhesion together with attenuated shear-induced aggregation due to reduced platelet GPIIbIIIa expression may impair the growth and stability of platelet thrombi [8, 22], and contribute to the decreased AUC10 in hemodialysis patients, even in the presence of elevated VWF. To define mechanisms involved more studies are, however, needed.

We studied effects of HD, but we could not observe any significant change in AUC10 values in response to HD overall. This finding is in agreement with a previous study by Eleftheriadis et al. who investigated the effects of HD on platelet function using another global platelet function test performed in platelet-rich plasma [24]. In our study, we performed a sub-group analysis comparing patients with normal and impaired platelet thrombogenicity, showing that four out of five patients with normal platelet thrombogenicity reduced their thrombogenicity after HD, but this was generally not the case in patients with already impaired platelet function prior to the start of the HD procedure. One possible explanation to the different changes in platelet thrombogenicity between these two groups could be that in ESRD patients with preserved platelet thrombogenicity, an intact GPIbα function and shear-induced platelet aggregation make platelets more vulnerable to HD, which is a procedure that have, as discussed above, been associated with reductions in GPIbα receptor expression [25]. This effect cannot be compensated for by increments in plasma VWF which under normal conditions is an important ligand-mediating platelet adhesion and aggregation under high shear. In patients with impaired platelet function already at the start of HD, other HD-related effects may be more important, such as elimination of uremic toxins or perhaps HD-induced hemoconcentration. The latter was not explicitly investigated in the present study as we did not measure hematocrit after HD. Of note, hemoconcentration following HD may lead to an enhancement in platelet thrombogenicity. Indeed, the positive effect on hemostasis through elevated hematocrit is well known in ESRD [26]. Although we have no data on blood cell count or hematocrit after HD it is likely that T-TAS01 would detect changes in hematocrit, since flow is laminar in the PL-chip and platelet thrombogenicity in T-TAS01 is dependent on flow and shear rate [27].

Different HD procedures may influence platelet function and other aspects of hemostasis and oxidative stress in various ways, as has e.g. been shown for dialysis membranes [4]. This may explain some of the discrepant data found in the literature of HD and its effects on hemostasis and platelet function.

The etiology of ESRD may be of importance when it comes to risk of thromboembolic complications, as shown recently in a large epidemiologic study [28]. Interestingly, two patients with intact platelet thrombogenicity, before as well as after hemodialysis, had diabetic nephropathy, the etiology associated with highest risk of thromboembolic complications [28]. It is well known that diabetes mellitus is associated with platelet hyperreactivity and abnormalities in platelet function, and hyperactive intracellular signaling pathways have repeatedly been reported in diabetes mellitus [29]. It is thus likely that various conditions or factors other than VWF influence platelet function and contribute to the variability in platelet thrombogenicity found in the present study. A global method which assesses the total thrombogenicity in a blood sample under flow may, therefore, be advantageous. Together with complementary and more “specific” methods, the mechanisms behind this between-patient variability in thrombogenicity could be studied in more detail.

Patients receiving HD are medically complex being a very heterogenous and diseased population, and clinical decision making is complicated in the patient with ESRD when it comes to antithrombotic treatment [30]. It has been emphasized that the bleeding risk in patients with ESRD should be thoroughly evaluated before starting antithrombotic treatment [31], thus leaving the clinician with difficult clinical judgements, balancing the risk of bleeding versus thrombosis. Of note, common bleeding risk scores developed in non-ESRD patients with atrial fibrillation patients perform poorly in ESRD patients [1]. Tools or methods that help the clinician in hard decisions to initiate or abstain from antithrombotic treatment in patients suffering from ESRD would be of great help. In this respect, T-TAS may be an option. It is a bed-side technique which uses whole blood, does not require any sample preparation, is easy to perform, and yields data that which can be instantaneously interpreted by the clinician.

In conclusion, the majority of ESRD patients in our study showed an impaired capacity to form platelet-dependent thrombi when assessed under high shear flow in whole blood with T-TAS01. To what extent a reduced platelet thrombogenicity as measured by T-TAS01 translates into a higher risk of bleeding in patients on HD, as has been reported in patients with cardiovascular disease or congenital platelet disorders [12], is unknown but deserves to be investigated in future studies. Indeed, tools that can help in risk stratification in ESRD patients, weighing risk of thrombosis vs bleeding, would be of great help, especially in HD patients as they paradoxically have an increased risk of both bleeding and thrombotic complications.

### Limitations

In this study, we compared VWF-related parameters and shear-based platelet thrombus formation in pre- and post-hemodialysis blood. However, we did not examine platelet aggregation or platelet membrane glycoprotein expression. A better understanding of the impact of hemodialysis on primary hemostasis and platelet function, including platelet-dependent thrombogenicity, requires an evaluation of these factors in much larger and adequately powered studies, and preferably in combination with methods used to study specific mechanisms, e.g. flow cytometry.

The cut-off for identifying impaired platelet function (AUC10 < 260) was obtained in a population of healthy individuals in the United States [14]. This cut-off should preferably be corroborated also in a population of healthy Serbian individuals.
